# Case Report: Traumatic obturator hip dislocation with subtrochanteric fracture in an 8-year-old child

**DOI:** 10.3389/fsurg.2025.1531518

**Published:** 2025-02-14

**Authors:** Shuming Huang, Kanghao Fang, Hailin Xing, Shuhua Lan, Quanzhou Wu

**Affiliations:** Department of Orthopaedic Surgery, The Fifth Affiliated Hospital of Wenzhou Medical University; Lishui Municipal Central Hospital, Lishui, Zhejiang, China

**Keywords:** hip dislocation, femoral fractures, pediatrics, hip fractures, traumatic, obturator

## Abstract

**Background:**

Obturator-type hip dislocation is a rare condition in pediatric patients, with the simultaneous occurrence of an ipsilateral subtrochanteric femoral fracture being exceptionally uncommon. Although various treatment methods have been described for hip dislocation or subtrochanteric femoral fractures, managing these injuries remains challenging due to the potential risk of femoral head avascular necrosis and concerns about fixation stability.

**Case presentation:**

An 8-year-old boy sustained a traumatic obturator hip dislocation and an ipsilateral subtrochanteric fracture following a fall from a height. Closed manual reduction of the hip dislocation was performed under general anesthesia, followed by internal fixation of the fracture using an adult proximal humerus locking plate. After 3 years of follow-up, the patient showed complete fracture healing and achieved a full, painless range of hip motion without any complications.

**Conclusion:**

This case describes a rare instance of traumatic obturator-type hip dislocation with an ipsilateral subtrochanteric femoral fracture in a child. It highlights an effective treatment approach for managing this complex injury.

## Introduction

Traumatic hip dislocation is rare in pediatric patients. In children, hip dislocation is classified into three types based on the position of the femoral head: anterior (pubic), anterior-inferior (obturator), or posterior dislocation (1). Obturator-type hip dislocation in pediatric orthopedics and traumatology has been infrequently reported (2–4). Concomitant ipsilateral subtrochanteric femoral fracture is even rarer, with only one case described in the English literature (1). This report presents a pediatric case of high-energy trauma caused by a fall from height, resulting in traumatic obturator-type hip dislocation and an ipsilateral subtrochanteric femoral fracture.

## Case description

**Figure 1:**
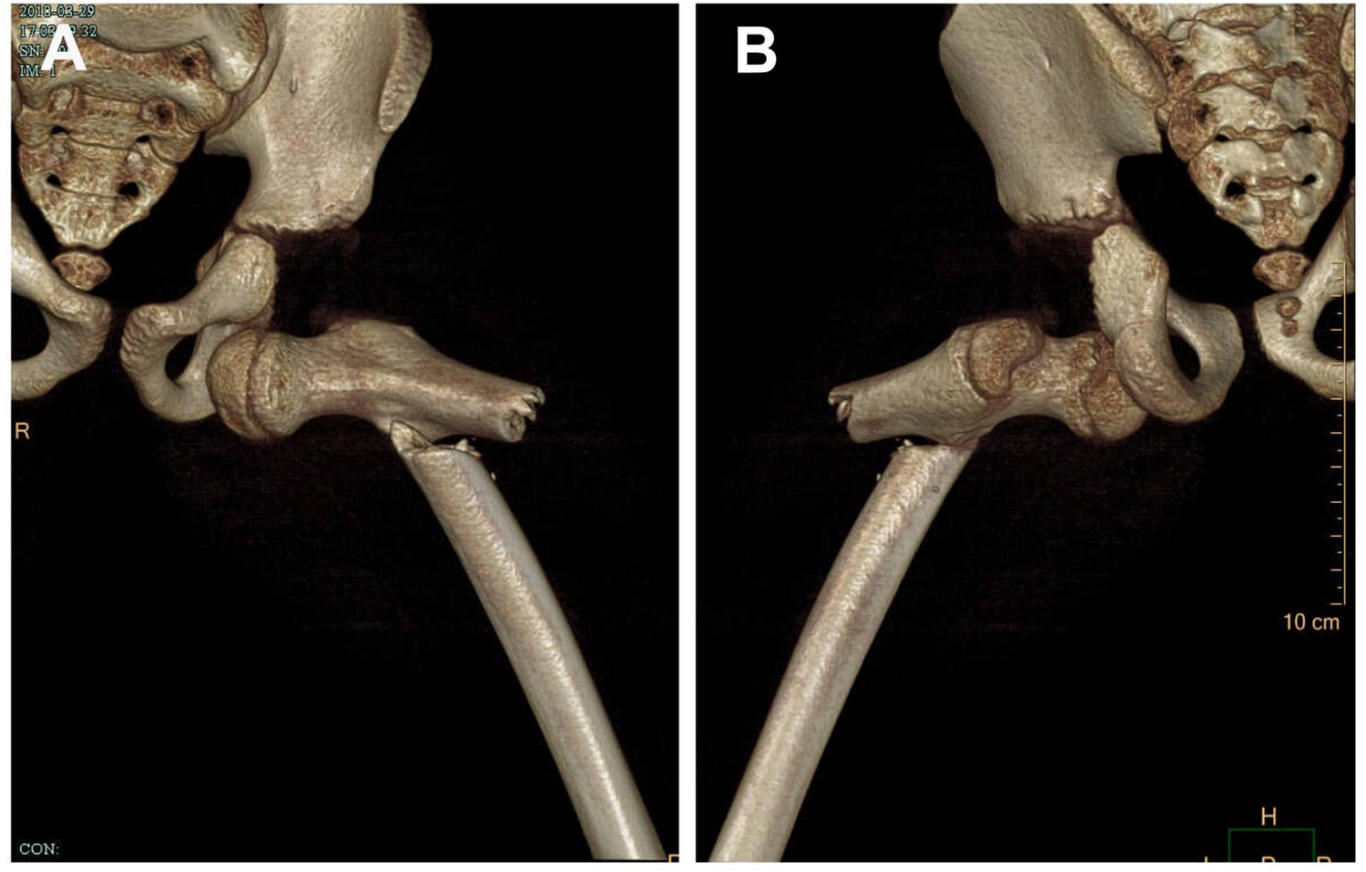
Three-dimensional CT scan of the hip of an 8-year-old child. **(A)** Anterior observation. **(B)** Posterior observation.

An 8-year-old boy presented to our emergency department 4 h after falling from a height of 8 m. His height was 1.28 m, and his body mass index (BMI) was 17.70. On admission, he complained of left hip pain, difficulty lifting the left lower limb, and an inability to bear weight. He exhibited a mildly altered level of consciousness, with a Glasgow coma score of 13. Clinical examination revealed left facial and periorbital edema and ecchymosis, along with congealed blood in the nostrils but no cerebrospinal fluid leakage. Further evaluation showed swelling and tenderness in the left hip and thigh, with hip deformity in abduction, external rotation, and flexion accompanied by shortening of the left lower limb. No neurovascular deficits or open wounds were noted in either lower limb. There was no history of joint laxity or prior dislocations. Routine blood tests showed: white blood cell count 13.0 × 10⁹/L, red blood cell count 3.25 × 10¹²/L, hemoglobin 96 g/L, and platelet count 218 × 10⁹/L. Other blood chemistry results, including liver and kidney function, electrolyte levels, and coagulation profiles, were within normal limits. A focused assessment with sonography for trauma (FAST) revealed no evidence of thoracoabdominal or pericardial fluid. Computed tomography (CT) of the left hip and femur confirmed an obturator-type hip dislocation with an ipsilateral subtrochanteric femoral fracture without fractures in the adjacency epiphysis (Figure 1). Additional CT findings included mild craniocerebral contusion, left maxillofacial and periorbital fractures, and a right occipital fracture. The injury severity score (ISS) was 22.

**Figure 2:**
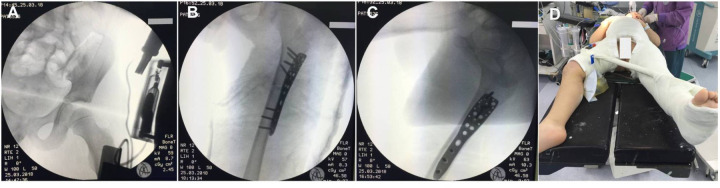
Intraoperative x-rays and postoperative casting for immobilization. **(A)** Anteroposterior radiograph of the left hip showed a reduced hip joint. **(B,C)** Intraoperative anteroposterior and lateral x-rays revealing fracture and dislocation reduction and fixation with an adult proximal humerus locking plate and screws. **(D)** A one-and-a-half-leg hip spica cast was performed immediately after surgery.

After confirming the absence of surgical contraindications or signs of hemodynamic shock, a gentle closed reduction maneuver was performed for hip dislocation within 2 h of admission under general anesthesia. The patient was positioned supine on a radiolucent orthopedic table. A surgeon grasped the flexed knee with one hand, maintaining continuous longitudinal traction on the femur, while manually repositioning the femoral head with direct palm pressure with the other hand. An assistant stabilized the pelvis. Post-reduction radiographs confirmed a congruent and stable hip joint (Figure 2A). The femoral fracture was treated with open reduction and internal fixation. The patient was repositioned in the right lateral decubitus position. A routine lateral incision was made, and the fracture was exposed by separating the vastus lateralis from the fascia lata. The fracture was stabilized with an adult proximal humerus locking plate system, which provided multiple screw fixations in the proximal segment. This system was selected due to the lack of availability of a pediatric multi-angle fixed hip plate. The plate's shape and size ensured compatibility with the proximal femoral anatomy avoiding any damage to the proximal femoral physis (Figures 2B,C). Finally, a one-and-a-half-leg hip spica cast was applied (Figure 2D).

**Figure 3:**
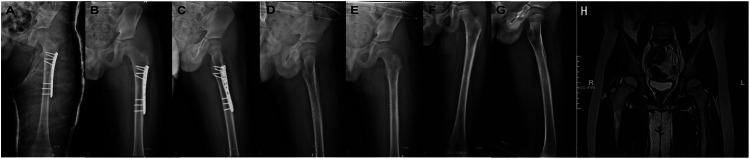
Postoperative radiographs and magnetic resonance imaging of the left hip and thigh. **(A)** Radiograph on postoperative day 2 showed an adequate reduction of the hip joint and subtrochanteric fracture with an excellent implant placement. **(B–E)** Fracture healing was observed before and after the removal of internal fixation (16 months after the primary surgery). **(F,G)** No signs of osteonecrosis, early closure of the epiphyseal plate, heterotrophic ossification, and osteoarthritis were observed on the anteroposterior and lateral radiographs at 3 years of follow-up after injury. **(H)** MRI of the hip at 3-year follow-up after injury showed no signs of osteonecrosis.

**Figure 4:**
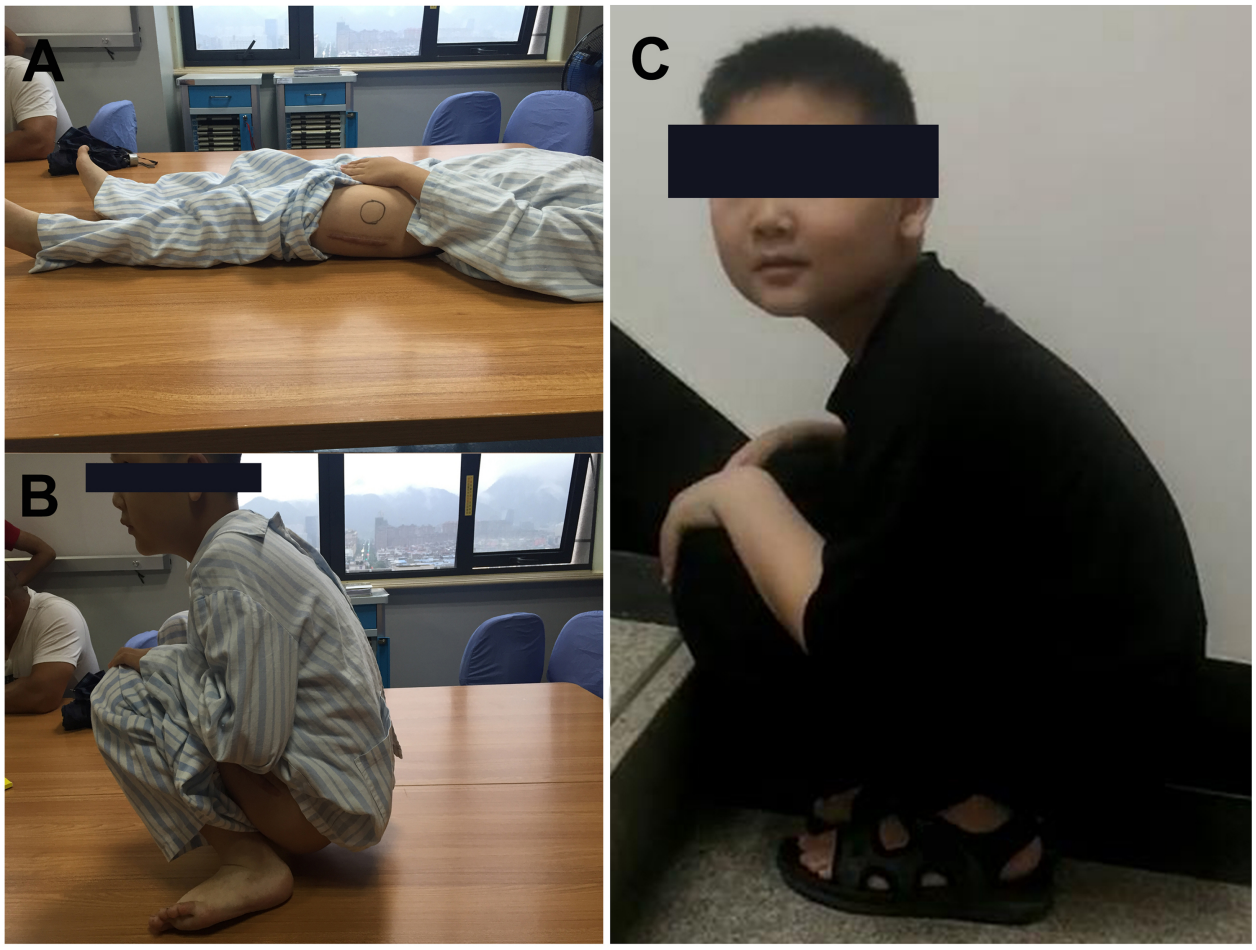
The functional outcome was satisfactory. **(A,B)** A full ROM of the hip was assessed before hardware removal. **(C)** At the time of the 3-year follow-up visit after injury.

Postoperative radiographs confirmed adequate reduction of the hip joint, proper alignment of the subtrochanteric fracture, and excellent implant placement (Figure 3). Additionally, a postoperative full-length anteroposterior femur radiograph and preoperative femoral CT confirmed the fracture location within 10% of the total femoral length below the lesser trochanter, meeting the definition of a subtrochanteric femoral fracture as described by Pombo and Shilt (5). At 4 weeks post-injury, the cast was removed to allow mobilization exercises. Fracture union was achieved by 8 weeks postoperatively. Partial weight-bearing walking with crutches was initiated at 2 months, progressing to full weight-bearing walking at 3 months post-surgery. The fixation was removed 16 months after the initial surgery. During follow-up, no complications, such as infection, re-dislocation, fixation failure, or vision issues, were observed. At the last follow-up (3 years postoperatively), the patient demonstrated a satisfactory functional outcome with a full range of motion in the left hip and knee joints. He returned to his previous activity level without pain or gait abnormalities (Figure 4). No clinical or radiological evidence of leg length discrepancy, premature epiphyseal plate closure, heterotrophic ossification, post-traumatic arthritis (PTA), or avascular necrosis (AVN) was identified (Figure 3).

## Discussion

Traumatic hip dislocation in children and adolescents is rare, comprising less than 5% of all hip dislocations. Anterior dislocations account for approximately 10% of these cases based on the direction of the dislocation (6). Closed manual reduction under anesthesia is successful in most cases of hip dislocation. However, open reduction may be required for irreducible dislocations, non-concentric reductions caused by intra-articular osteochondral or soft tissue fragments, or dislocations associated with acetabular or femoral head fractures (1, 6). The choice of anterior or posterior surgical approach depends on the direction of the dislocation, the need to address instability factors, and the management of associated fractures (7). Reports of obturator hip dislocation combined with concurrent injuries to adjacent structures, such as the femoral head, neck, greater trochanter, or shaft, are limited in the English literature, as summarized in Table 1 (1, 8–12).

**Table 1:** Studies in literature reporting cases of hip anterior obturator type dislocation and ipsilateral femoral fracture.

First author (year of publication)	Years\sex	Side	Injury mechanism	Type of dislocation/fracture	Hip reduction method	Fracture surgery method	Complication	Following time and function
Malkawi, 1982 (1)	7/M	R	Car accident	Obturator/ Bilateral transverse subtrochanteric fractures	Invasive	ORIF with compression plate of Ipsilateral fracture, conservative treatment (skin traction) of contralateral fracture	Femoral head showed normal appearance; no clinical complaints	1 year; no mention of function status
Yamamoto, 2004 (8)	2/F	R	Car accident	Obturator/ Transverse shaft	Manual	Conservative treatment with Bryant traction for 4 weeks, followed by 3 week of cast immobilization with pelvic band	No clinical complaints; no AVN abnormal signals	4.5 years; able to engage in usual activities; normal ROM and gait
Arjun, 2016 (9)	11/M	L	Bike accident	Obturator/ Transverse shaft	External fixator closed reduction	Closed reduction and internal fixation with intramedullary interlocking nail, hip immobilized with skin traction for 3 weeks	No signs of hip dysplasia or AVN or coxa magna	6 months; near-normal hip ROM,; returned to his earlier level of activity
Durand, 2018 (10)	15/M	L	Bike accident	Obturator/ Pipkin type 1 head	Manual	Conservative treatment, no weight bearing for 6 weeks (not allowed to bear weight for 6 weeks), hip flexion over 60 degrees forbidden	AVN after 2 months, treated with femoral head drilling and stem cell injection (perform a drilling of the femoral head and stem cell injection)	1 year; no pain or lameness; practice BMX at a high level again; the hip flexion was 120°; external and internal rotations were 30°–0°–20° vs. 30°–0°–25°
Cao, 2019 (11)	7/M	R	Car accident	Obturator/ Open injury of proximal thigh and transverse shaft fracture + Contralateral proximal and distal fracture	The hip reduced hip dislocation—and debridement after 10 + hours	1 week posttrauma, ipsilateral femur fixed with a straight plate; contralateral proximal femur ORIF with PHILOS, distal femur closed reduction and fixed with Kirschner wires	AVN of the femoral head after 8 months	8 months; free of pain; complete ROM of hip and knee
Khalifa, 2019 (12)	13/M	R	Tractor wheel crush accident	Open anterior dislocation/ Greater trochanter + Contralateral diaphyseal fracture	Cleaned and debrided + Manual reduction	Conservative treatment of ipsilateral femur; ORIF + DCP plate of contralateral femur	Periarticular ossification at 1 year; Advanced AVN at 3 years	3 years, poor function; limited hip ROM (flexion 100°, extension 10°, abduction 20°, adduction 108, internal rotation 20°, external rotation 30°)
Present report	8/M	L	Fall from height accident	Obturator/ Ipsilateral subtrochanteric fracture	Manual	ORIF with PHILOS	No signs of hip dysplasia, AVN, or coxa magna; no pain or lameness	3 years; full ROM of hip and knee

F, female; M, male; Yrs, years; L, left; R, right; ORIF, open reduction and internal fixation; PHILOS, proximal humerus internal locking system; DCP, dynamic compression plate; AVN, avascular necrosis; ROM, range of motion.

Violent reduction techniques for hip dislocation may result in periarticular fractures, epiphyseal separation, or AVN. Thus, closed reduction without sedation or general anesthesia in the emergency setting is not recommended. Currently, there is no consensus regarding the optimal reduction method for hip dislocation or the appropriate duration of non-weight-bearing after reduction. Anterior hip dislocation with a femoral shaft fracture can be reduced by sustained longitudinal traction using an external fixator system with Schanz screws in the proximal femur (9) or gentle traction to the lower leg (8). In this case, the left hip dislocation was successfully reduced within six hours of injury using gentle traction of the lower limb, with the femoral head pushed under general anesthesia during fluoroscopy. The hip was then immobilized with a spica cast for 4 weeks, consistent with previous recommendations (13) to prevent recurrent dislocation and AVN associated with early weight-bearing.

Titanium elastic nails (TENs) are currently a widely used treatment for femoral shaft fractures in children and young adolescents. They offer several advantages, including reduced intraoperative blood loss, preservation of the fracture hematoma, shorter healing times, and earlier weight-bearing (14). However, in proximal third femur fractures, TENs are associated with a 22% complication rate, and achieving reduction during surgery can be challenging (15). Compared to TENs, open reduction and internal fixation with locking plates for pediatric subtrochanteric femur fractures result in reduced x-ray exposure, better outcome scores, and a lower overall complication rate (16, 17). Displaced subtrochanteric femoral fractures pose significant challenges due to their proximity to the greater trochanter epiphyseal plate, short proximal metaphyseal fragments, limited remodeling capacity, and inherent instability (16). Although the fracture in this case was not comminuted, it was considered unstable due to its subtrochanteric location. TENs may not have offered the necessary rigidity to withstand the biomechanical demands of this region, increasing the risk of malalignment or delayed union. For these reasons, open reduction and internal fixation with a locking plate were chosen as the surgical treatment in this case to ensure rotational stability and minimize complications such as shortening or malalignment.

When pediatric proximal femoral locking plates are unavailable during emergency surgery, an adult proximal humerus locking plate may serve as an effective alternative for treating pediatric subtrochanteric femoral fractures. This plate provides rigid fixation with multiple locking screw options in various directions for the proximal segment while avoiding interference with the epiphyseal growth plate. It enhances angular stability and permits distal fixation using longer plates (18). The adult proximal humerus locking plate was initially reported as a treatment for an atrophic nonunion of a subtrochanteric femur fracture in an 11-year-old boy, demonstrating its ability to adapt to the proximal femoral anatomy and achieve strong fixation (19). In this case, additional fixation of the femoral neck was not required due to the specific fracture characteristics. However, for more complex fractures, the plate can be adjusted to accommodate additional screws, allowing for long screws to be inserted into the femoral neck to optimize fixation. These implants have shown good outcomes and stability in pediatric subtrochanteric fractures (20). The use of an adult proximal humerus locking plate for subtrochanteric fractures in children offers a viable alternative to pediatric hip plates for highly proximal femoral fractures and should be considered as part of the surgical armamentarium.

## Conclusions

This report presents a rare polytrauma case of traumatic obturator-type hip dislocation with a subtrochanteric fracture of the ipsilateral femur in a child. The subtrochanteric fracture was successfully treated with open reduction and internal fixation using an adult proximal humerus locking plate after closed manual reduction of the hip dislocation. This case highlights an effective alternative treatment for managing such complex injuries.

## Data Availability

The original contributions presented in the study are included in the article/Supplementary Material, further inquiries can be directed to the corresponding author.

## References

[1] Malkawi H. Traumatic anterior dislocation of the hip with fracture of the shaft of the ipsilateral femur in children: case report and review of the literature. J Pediatr Orthop. (1982) 2(3):307–11. 10.1097/01241398-198208000-00013 7130390

[2] Avery DM 3rd Carolan GF. Traumatic obturator hip dislocation in a 9-year-old boy. Am J Orthop (Belle Mead NJ). (2013) 42(9):E81–3. 24078972

[3] Ahmad S Devkota P Mamman KG. Traumatic anterior dislocation of hip in a child—case report. Malays Orthop J. (2015) 9(1):30–1. 10.5704/MOJ.1503.003 28435593 PMC5349345

[4] Gupta V Kaur M Kundu ZS Kaplia A Singh D. Traumatic anterior hip dislocation in a 12-year-old child. Chin J Traumatol. (2013) 16(2):122–5. 10.3760/ma.j.issn.1008-1275.2013.02.012 23540904

[5] Pombo MW Shilt JS. The definition and treatment of pediatric subtrochanteric femur fractures with titanium elastic nails. J Pediatr Orthop. (2006) 26(3):364–70. 10.1097/01.bpo.0000203005.50906.41 16670550

[6] Braun ME Loose O Schmittenbecher P Schneidmüller D Strüwind C Schwerk P Epidemiology and injury morphology of traumatic hip dislocations in children and adolescents in Germany: a multi-centre study. Eur J Trauma Emerg Surg. (2023) 49(4):1897–907. 10.1007/s00068-023-02280-2 37261461

[7] Benedick A Lopas L Daley E Jang Y. Traumatic hip dislocation: pediatric and adult evaluation and management. J Am Acad Orthop Surg. (2024) 32(14):637–46. 10.5435/JAAOS-D-23-01013 38713755

[8] Yamamoto K Ko M Masaoka T Shishido T Imakiire A. Traumatic anterior dislocation of the hip associated with ipsilateral femoral shaft fracture in a child: a case report. J Orthop Surg (Hong Kong). (2004) 12:126–32. 10.1177/230949900401200123 15237135

[9] Arjun RH Kumar V Saibaba B John R Guled U Aggarwal S. Ipsilateral obturator type of hip dislocation with fracture shaft femur in a child: a case report and literature review. J Pediatr Orthop Part B. (2016) 25:484–8. 10.1097/BPB.0000000000000324 27128394

[10] Durand Y Bruyère C Saglini M Michel-Traverso A. Traumatic obturator hip dislocation with marginal femoral head fracture in a 15-year-old adolescent: a high-energy trauma-a case report and a review of the literature. Case Rep Orthop. (2018) 2018:7268032. 10.1155/2018/7268032 30123600 PMC6079582

[11] Cao Z Zhu D Li C Li YH Tan L. Traumatic anterior hip dislocation with associated bilateral femoral fractures in a child: a case report and review of the literature. Pan Afr Med J. (2019) 32:88. 10.11604/pamj.2019.32.88.17497 31223379 PMC6560987

[12] Khalifa MA Alaya Z Hassini L Bouattour K Osman W Ben Ayèche ML. Ipsilateral open anterior hip dislocation and avulsion fracture of the greater trochanter: an unusual case report. Arch Pediatr. (2019) 26(7):422–5. 10.1016/j.arcped.2019.09.005 31630902

[13] Sulaiman AR Munajat I Mohd EF. Outcome of traumatic hip dislocation in children. J Pediatr Orthop B. (2014) 23(2):204–5. 10.1097/BPB.0000000000000007 24447940

[14] Zhang Y Xue Y Zhao M Chen X Gao Q. Titanium elastic nails vs locking plate in pediatric subtrochanteric femur fractures: a systematic review and meta-analysis. Front Pediatr. (2023) 11:1114265. 10.3389/fped.2023.1114265 36937961 PMC10020654

[15] Ho CA Skaggs DL Tang CW Kay RM. Use of flexible intramedullary nails in pediatric femur fractures. J Pediatr Orthop. (2006) 26(4):497–504. 10.1097/01.bpo.0000226280.93577.c1 16791069

[16] Başaran SH Bilgili MG Erçin E Bayrak A Öneş HN Avkan MC. Treatment and results in pediatric traumatic hip dislocation: case series and review of the literature. Ulus Travma Acil Cerrahi Derg. (2014) 20(6):437–42. 10.5505/tjtes.2014.52822 25541924

[17] Sanjay N Seenappa H Shanthappa AH Kumar K V. Functional outcome of pediatric subtrochanteric fractures treated with a Titanium elastic nailing system (TENS) versus plating. Cureus. (2023) 15(6):e40036. 10.7759/cureus.40036 37425582 PMC10324435

[18] Danişman M Özdemir E Dursun G Ayvaz M Yilmaz G. An alternative fixation option for subtrochanteric femur fractures in children: adult proximal humerus plate. J Pediatr Orthop. (2022) 42(8):e828–32. 10.1097/BPO.0000000000002207 35834366

[19] Cortes LE Triana M Vallejo F Slongo TF Streubel PN. Adult proximal humerus locking plate for the treatment of a pediatric subtrochanteric femoral nonunion: a case report. J Orthop Trauma. (2011) 25(7):e63–7. 10.1097/BOT.0b013e3181f8d9c3 21577158

[20] Gogna P Mohindra M Verma S Thora A Tiwari A Singla R. Adult proximal humerus locking plate for fixation of paediatric subtrochanteric fractures. Musculoskelet Surg. (2014) 98(3):189–94. 10.1007/s12306-013-0310-z 24402680

